# Insulin-stimulated mTOR activation in peripheral blood mononuclear cells associated with early treatment response to lithium augmentation in rodent model of antidepressant-resistance

**DOI:** 10.1038/s41398-019-0434-5

**Published:** 2019-03-15

**Authors:** Adam J. Walker, J. Blair Price, Kristin Borreggine, Shari L. Sutor, Andrea Gogos, Jane A. McGillivray, Mark A. Frye, Susannah J. Tye

**Affiliations:** 1 Department of Psychiatry and Psychology, Mayo Clinic, Rochester, MN USA; 2 School of Psychology, Deakin University, Burwood, VIC Australia; 3 IMPACT Strategic Research Centre, School of Medicine, Deakin University, Geelong, VIC Australia; 4 Hormones in Psychiatry Laboratory, Florey Institute of Neuroscience and Mental Health, Parkville, VIC Australia; 5 School of Medicine, Deakin University, Waurn Ponds, VIC Australia; 6 Department of Psychiatry, University of Minnesota, Minneapolis, MN USA; 7 Department of Molecular Pharmacology and Experimental Therapeutics, Mayo Clinic, Rochester, MN USA; 8 Queensland Brain Institute, The University of Queensland, St Lucia, QLD Australia

## Abstract

Lithium has been shown to have some therapeutic efficacy as an adjunctive treatment for intractable forms of major depression. Activation of mammalian target of rapamycin (mTOR) and inhibition of glycogen synthase kinase-3β (GSK3β) have been implicated in its putative mechanisms of action. These proteins are integral components of the insulin signaling pathway, which may serve as a critical co-regulator of drug action. Utilizing an animal model of tricyclic antidepressant resistance, we investigated the relationship between insulin signaling and antidepressant response to lithium augmentation. Pre-treatment with adrenocorticotropic hormone (ACTH 100 µg/day i.p.) for 14 days effectively blocked the immobility-reducing effects of an acute dose of imipramine (10 mg/kg i.p.) in the forced swim test (FST). Lithium augmentation (100 mg/kg i.p.) rescued the antidepressant-like effects of imipramine in this model. Total and phosphorylated (*p*) levels of protein kinase B (Akt), mTOR, and GSK3β protein were quantified in the infralimbic cortex (ILPFC) following FST stress via Western blot. Levels of mTOR and *p*mTOR were further quantified in isolated peripheral blood mononuclear cells (PBMCs) following insulin stimulation (10 mg/mL for 5 min) via ELISA. Elevated levels of phosphorylated insulin signaling proteins were present in the ILPFC of ACTH-pretreated animals that received both imipramine and lithium, together with a concurrent increase in mTOR activation in PBMCs. Large correlations were observed between immobility time and insulin-stimulated mTOR levels in PBMCs. We propose that PBMC insulin challenge may be a useful probe for predicting antidepressant response to lithium administration, and potentially other therapies acting via similar mechanisms of action.

## Introduction

Suboptimal response to antidepressant treatments remains a major challenge in psychiatry. Indeed few (if any) useful biomarkers exist to predict early treatment efficacy^1^. The identification of acute physiological correlates for early antidepressant response could potentially lead to their utilization as biomarkers. Such biomarkers could provide insight into discreet pathophysiological processes contributing to the antidepressant response. Such processes, in turn, have potential to curtail the extended misapplication of ineffective treatments currently necessitating prolonged periods of observation. Although many preclinical models of depression exist, few models focus on improving our understanding of behavioral resistance to antidepressants.

Aberrant activation of the hypothalamic-pituitary adrenal (HPA) stress axis is commonly reported in both unipolar and bipolar depressed patients^2,3^ and is associated with sub-optimal treatment responses, increased symptom severity, and poorer remission rates^3,4^. Chronic stimulation of the HPA axis via administration of adrenocorticotropic hormone-(1–24) (ACTH) blocks the immobility-reducing effects of imipramine in the forced swim test (FST)^5–9^, a well-established behavioral screen for antidepressant efficacy in rodents. Kitamura and colleagues^6^ previously reported that lithium co-administration (100 mg/kg p.o.) in this model could rescue the antidepressant response to imipramine (10 mg/kg) in the FST.

**Fig. 1:**
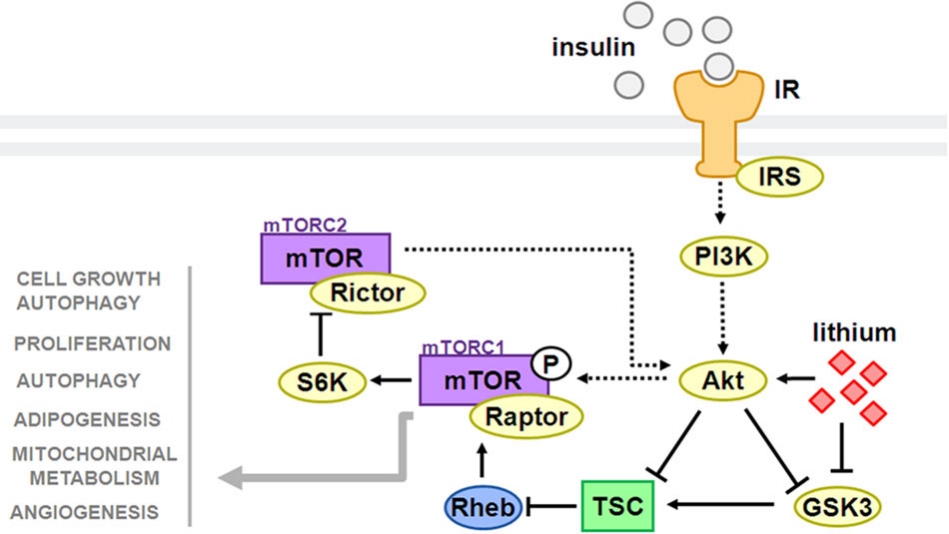
Simplified schematic diagram of the effects of lithium on insulin signaling. Lithium inhibits GSK3β both directly and indirectly (via Akt facilitation), which can have subsequent downstream effects on mTOR activity

Lithium is effective in both manic and prophylactic treatment, and remains the gold standard mood stabilizing medication for bipolar patients^10,11^. This mood stabilizer has also been shown to improve therapeutic efficacy and extend remission when applied as an adjunctive treatment for intractable forms of major depression (reviewed in refs. ^11,12^); however, there remains a proportion of patients that do not receive therapeutic benefit from lithium^13,14^. Our understanding of lithium’s therapeutic mechanism, and consequently, our ability to rationally identify biomarkers predictive of lithium’s therapeutic efficacy, is still limited. One mechanism believed to contribute to lithium’s therapeutic action is the inhibition of glycogen synthase kinase-3β (GSK3β), a key mediator of energy metabolism within the insulin signaling pathway^15^. Importantly, in the context of insulin signaling, GSK3β is known to interact with mammalian target of rapamycin (mTOR). mTOR serves an integral regulatory role in cellular energy, growth and plasticity; responding to various environmental stressors and modulating gene transcription/translation, apoptotic processes, and synapse formation in accordance with energy availability^16–18^. Via these actions, mTOR activation has been implicated in the novel antidepressant-like effects of ketamine and other antidepressants (for review, see ref. ^19^). Together with protein kinase B (Akt), mTOR and GSK3β form an integral portion of the insulin signaling pathway, and are important for cellular energy regulation and metabolism (see Fig. 1^20,21^).

Post-mortem human brain studies comparing unipolar MDD patients to healthy controls have demonstrated abnormalities in both mTOR and GSK3β levels in the prefrontal cortex (PFC)^22,23^. Reduced levels of phosphorylated GSK3β have also been previously reported in the peripheral blood mononuclear cells (PBMCs) of bipolar patients, correlating with symptom severity^24^. Additionally, Machado-Vieira and colleagues^25^ reported lowered *Akt1* and *mTOR* mRNA expression in the blood of bipolar patients during depressive episodes, where changes in *Akt1* expression following lithium treatment were associated with clinical improvement.

Given that a direct and interdependent relationship exists between the neuroendocrine response to stress and energy regulation and metabolism, we propose that long-term aberrant HPA activity may impair metabolic functions under stress, contributing to poor antidepressant efficacy at the molecular and behavioral level. Such metabolic consequences may be particularly pertinent for antidepressant action in regions of the depression network known to be hypermetabolic, such as the infralimbic cortex (ILPFC). The ILPFC region of the medial PFC plays a critical role in regulating behavioral responses to stress, particularly in the FST. The ILPFC is considered to be the rodent homolog of Brodmann’s area 25 (BA25) in humans^26,27^. Notably, BA25 is metabolically overactive in patients with depression; and normalizing this metabolic disturbance has been associated with antidepressant responses across multiple modalities^28,29^. Failure to normalize this metabolic hyperactivity through antidepressant treatment is, by contrast, associated with poor clinical outcomes in treatment-resistant depression.

In this study, we sought to verify that lithium augmentation restores the antidepressant-like effects of imipramine in ACTH-pretreated animals as measured by the FST and to determine if such effects were associated with the expression of insulin signaling pathway proteins. ACTH pre-treatment is expected to block the typical immobility-reducing effects of imipramine in the FST, while a reduction in immobility duration and corresponding increase in active coping strategies, such as climbing is expected following co-administration of lithium and imipramine in ACTH animals.

Following behavioral experiments, we quantify levels of insulin signaling proteins (Akt, mTOR, and GSK3β) in the ILPFC. Consistent with clinical data described above, we expect protein expression in the ILPFC of ACTH animals to reflect a deficit in this signaling pathway, which will be rescued by imipramine when co-administered with lithium, but not when delivered as a monotherapy. Finally, we aim to affirm the potential for insulin-mediated signaling in PBMCs to serve both as a behaviorally relevant proxy marker of insulin signaling in the brain, and as a potential peripheral marker for early treatment response to lithium.

## Experimental procedures

### Animal treatments

Male albino Wistar rats (*n* = 60) were used in this study, weighing 250–350 g at the time of testing. Animals were housed individually in a room with controlled temperature (20–22 °C) on a 12 h light–dark cycle (lights: on 07:00; off 19:00). Food and water were available ad libitum. Animals entered the study at 5 weeks of age following a 3-day acclimatization period, and completed testing in their seventh week, at which point they were sacrificed. All procedures were carried out in accordance with institutional guidelines for ethical animal care and use.

### Drugs

The drugs used in this study included: ACTH-(1–24) (AnaSpec, San Jose, CA, USA), 100 μg/day dissolved in distilled water; imipramine hydrochloride 10 mg/kg (Sigma-Aldrich, St. Louis, MO, USA); lithium chloride 100 mg/kg (Sigma-Aldrich); control vehicle 0.9% saline (Fisher Healthcare, Hanover Park, IL, USA); and FatalPlus^®^ (Vortech Pharmaceuticals, Dearborn, MI, USA), (constituents: pentobarbital sodium 390 mg/mL; propylene glycol 0.01 mg/mL; ethyl alcohol 0.29 mg/mL; benzyl alcohol (preservative) 0.20 mg/mL) 0.70 cc. Drugs were all delivered via intraperitoneal (i.p.) injection.

### Experimental procedure

**Fig. 2:**
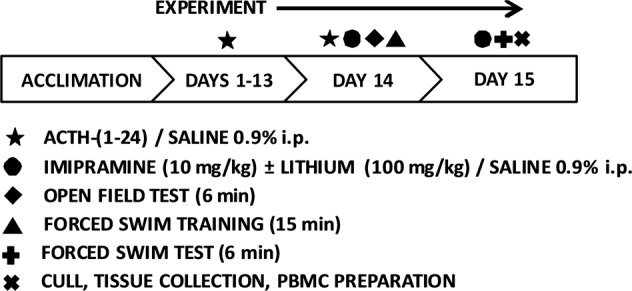
Schematic diagram depicting of the timeline for behavioral experiments. A key corresponding to the symbols used is provided below the timeline

This study implemented a treatment protocol described previously with minor modifications (see ref. ^9^). Briefly, following acclimatization, male Wistar rats were randomly assigned to receive daily injections of either ACTH-(1–24) 100 μg/day or saline (0.9%) for 14 days. On treatment day 14 the open field test (OFT) was administered and 2 h post-test animals received their initial 15 min forced swim stress exposure. A final 6 min FST was conducted on day 15. Behavioral data was recorded for analyses. Animals were administered either imipramine hydrochloride 10 mg/kg, imipramine hydrochloride 10 mg/kg with lithium chloride 100 mg/kg, or control vehicle saline (0.9%) on day 14 and 15, 30 min prior to the OFT and FST, respectively. Animals were sacrificed 30 min after FST on day 15 via anesthetic overdose of pentobarbital sodium (0.7 cc Fatal-Plus^®^). Brains were harvested and cardiac blood samples were also collected. Samples were frozen on dry ice and stored at −80 °C until use. A schematic diagram of the experimental timeline is depicted in Fig. 2.

### Behavioral testing

#### Open field test

The OFT was implemented to examine ambulatory/locomotor behaviors in response to treatments. Animals were each placed in the central zone of an open field arena (60 cm length × 60 cm width × 60 cm height), and allowed to move freely for 6 min. Behaviors were recorded by video camera. Data were analyzed using the behavioral analysis package TopScan (CleverSys Inc., Reston, VA, USA). Behaviors of interest included: *distance traveled*, *mean velocity of travel*, and *time spent in central region of arena*.

#### Forced swim test

The FST is a well-established screening tool for evaluating antidepressant efficacy, with robust predictive validity^30^. The forced swim apparatus consisted of clear plexiglass cylindrical tanks (45 cm height × 20 cm diameter) filled with tap water (23 °C) to a depth of 30 cm. Animals were first exposed to 15 min learning trial, conducted 2 h after the completion of the OFT on day 14. A 6 min test session was then conducted on the subsequent day. Sessions were recorded, and analyzed using the behavioral analysis package ForcedSwimScan (CleverSys Inc.). Following ForcedSwimScan analysis footage was then deidentified and reanalyzed by hand in 1 s intervals for verification. As measured previously^9^, behaviors of interest included *immobility* (passive behavior), *swimming*, and *climbing* (active behaviors). The final 4 min of the FST were used for this analysis.

### Tissue collection

Following behavioral testing on day 15, animals were humanely euthanized by anesthetic overdose 30 min after completion of the FST. Cardiac blood samples were collected for PBMC isolation. The brain of each animal was extracted, frozen on dry ice, and stored at −80 °C until dissection.

### Western blotting

Brains were manually dissected on a ThermalTray™ LP (BioCision, Mill Valley, CA, USA) maintained at −20 °C using dry ice. Brain regions were identified using a rodent brain atlas (Figs. 9–13 in Paxinos and Watson^31^). Following dissection, ILPFC tissue was lysed in radioimmunoprecipitation assay (RiPA) lysis buffer for Western blotting. Protein concentration was determined by bicinchoninic acid (BCA) protein assay.

Equal amounts of ILPFC protein lysate were loaded and subjected to sodium dodecyl sulfate polyacrylamide gel electrophoresis (SDS–PAGE), before transfer to polyvinylidene fluoride (PVDF) membrane (Immobilon-P). Membranes were blocked for 2 h with tris-buffered saline solution with the detergent Tween^®^ 20 (TBST) containing 5% milk (or 5% bovine serum albumin (BSA) for phosphorylated antibodies), before being incubated at 4 °C with primary antibodies overnight. Antibodies used included: total Akt (pan), phospho-Akt (Ser^473^), GSK-3β, phospho-GSK-3β (Ser^9^), total mTOR, phospho-mTOR (Ser^2448^) (Cell Signaling Technology, Danvers, MA, USA); total β-actin (Sigma-Aldrich). Herein phosphorylated proteins will be prefixed with ‘*p*’.

The following day, blots were washed with TBST three times and incubated with anti-rabbit HRP-linked secondary antibody (Cell Signaling) for 45 min. Blots were then washed three more times with TBST and exposed to enhanced chemiluminescence (ECL) substrate. Band detection and densitometric analysis was conducted using Bio-Rad ChemiDoc™ imaging system. Readings were normalized to β-actin, and expressed as a ratio to cerebellar tissue (positive control).

### PBMC isolation and insulin challenge

#### Cell isolation and preparation

Dulbecco’s phosphate-buffered saline (DPBS) (1–4 mL) (Gibco Life Technologies, Rockvile, MD, USA) was added to a 10 mL heparinized tube (Monoject^®^: Kendall Healthcare, Mansfield, MA, USA) containing blood sample; added according to sample volume (2–8 mL), and mixed well with a pipette until homogenous. The blood/DPBS mixture was slowly added to a 15 mL conical polypropylene tube (BD Falcon™: BD Biosciences, Bedford, MA, USA) prefilled with 2 mL of Histopaque^®^ medium (solution containing polysucrose and sodium diatrizoate adjusted to a density of 1.077 g/mL) (Sigma-Aldrich), forming a layer atop it. Tubes were immediately centrifuged at 400 × *g* for 30 min facilitating the separation of the PBMCs from the plasma and erythrocytes. Excess plasma was then removed from the tube before the PBMC layer was collected with a 5 mL pipette.

The PBMCs were then deposited into T25 tissue culture flasks (BD Biosciences) filled with 5 mL Roswell Park Memorial Institute (RPMI) medium 1640 (Gibco Life Technologies) containing 10% fetal bovine serum (FBS), l-glutamine, 4-(2-hydroxyethyl)-1-piperazineethanesulfonic acid (HEPES) and penicillin/streptomycin solution. Flasks were incubated overnight at 37 °C 5% CO_2_ in a humidified tissue culture incubator.

The following day, the cells were harvested and centrifuged at 1200 rpm for 5 min at 4 °C. Media was then aspirated from the tube, leaving the cells in a pellet at the base of the tube. Cells were then resuspended in 2 mL media containing 5% dimethyl sulfoxide (DMSO) (ATTC, Manassas, VA, USA), and dispensed into 2 mL cryogenic vials (Corning Inc., Corning, NY, USA) in 1 mL aliquots, placed in a styrofoam holder and slow frozen in a −80 °C freezer.

#### Insulin challenge and enzyme-linked immunosorbent assays (ELISA)

Frozen PBMCs were thawed and left to recover in a humidified tissue culture incubator (37 °C; 5% CO_2_) overnight. The following morning, cells were centrifuged and then re-suspended in media without FBS. Re-suspended cells were then divided into two wells of a six-well plate and left to incubate, devoid of growth factors for 4 h at 37 °C to deplete any residual insulin.

The cells were then challenged with 10 mg/mL of insulin for 5 min (37 °C; 5% CO_2_). Following challenge, cells were then centrifuged and lysed using 50 mL of RIPA lysis buffer. Cellular debris was removed by centrifugation (14,000 rpm; 4 °C; 10 min) and the lysate was then dispensed into a new tube. Commercially available competitive ELISA kits were used to test concentrations of mTOR (Total) and *p*mTOR (Ser^2448^) (Cell Signaling). A BCA protein assay was not possible because of the limited measurable protein levels; therefore results were reported in relative arbitrary units and relative differences pre-versus post-insulin quantified.

### Statistical analyses

In all cases, the Shapiro–Wilk test of normality and Brown–Forsythe test for homogeneity of variance were utilized; these assumptions were met unless otherwise specified. Separate one-way analysis of variance (ANOVA) with subsequent Tukey’s honest significant difference (HSD) tests were used to analyze both behavioral and molecular data. One-way ANOVA is considered a robust test against violations of the normality assumption, in this case minor violations were noted. Where the assumption of homogeneity of variance was violated, data were analyzed using non-parametric Kruskal–Wallis one-way tests followed by Dunn’s multiple comparison tests. Linear regression analysis was used to evaluate the relationship between the protein levels and behavioral results. Calculations were performed using GraphPad Prism 6.0.

## Results

### Behavioral data

#### Open field test

Brown–Forsythe tests for homogeneity of variance were significant for *distance*: *F*(4,50) = 3.071, *p* = 0.0244; *velocity*: *F*(4,50) = 3.195, *p* = 0.0206; and *centre duration*: *F*(4, 50) = 32.88, *p* = 0.0319. As such, non-parametric tests were used.

**Fig. 3:**
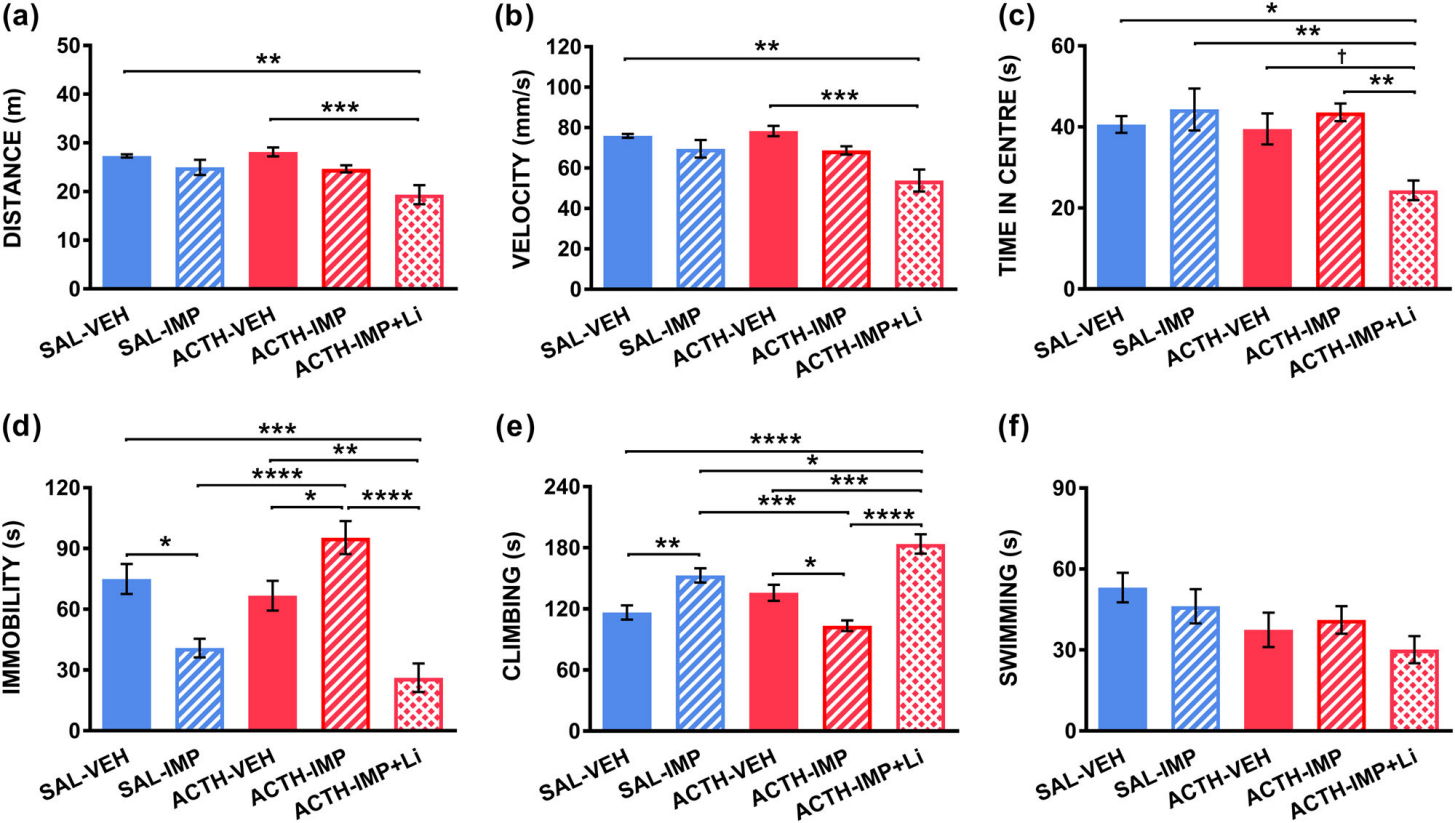
Lithium restores antidepressant-like effect of imipramine in FST. Treatment group means for (**a**) distance traveled (**b**) velocity of travel, and (**c**) time spent in center in the OFT; as well as (**d**) immobility duration, (**e**) climbing time and (**f**) swimming time in the FST (*n* = 10–12). Data are expressed as mean ± S.E.M; ^†^*p* < 0.08; *p < 0.05; **p < 0.01, ***p < 0.001, ****p < 0.0001

A one-way Kruskal–Wallis test unveiled a significant main effect of treatment on *distance* traveled in the OFT, *H*(4) = 19.06, *p* = 0.0008. Post-hoc Dunn’s tests indicated that the addition of lithium to imipramine treatment in ACTH pre-treated animals significantly reduced distance traveled compared to saline-control, and ACTH-control treatment groups (shown in Fig. 3a).

A one-way Kruskal–Wallis test also revealed a significant main effect of treatment on ambulatory *velocity*, *H*(4) = 19.13, *p* = 0.0007. Post-hoc Dunn’s tests suggested that ACTH animals treated with imipramine and lithium showed significantly lower velocity of travel when compared to saline-control, and ACTH-control animals (see Fig. 3b).

A one-way Kruskal–Wallis test also revealed a significant main effect of treatment on ambulatory *velocity*, *H*(4) = 19.05, *p* = 0.0019. Dunn’s post-tests identified that ACTH animals administered both imipramine and lithium spent significantly less time in the center region than animals in the saline-control, saline-imipramine, and ACTH-imipramine groups (shown in Fig. 3c).

Five animals were omitted from further analysis following their identification as outliers in the OFT, with ambulatory behavioral scores exceeding two standard deviations from the mean.

#### Forced swim test

One-way ANOVA exposed a significant main effect of treatment on *immobility* duration, *F*(4,50) = 14.954, *p* *<* 0.0001. Shapiro–Wilk normality test for ACTH-imipramine group was significant, *W* = 0.8213, *p* = 0.0179. Post-hoc Tukey’s HSD tests multiple comparisons revealed no significant difference between control saline-control and ACTH-control groups. Saline animals administered imipramine showed a significant reduction in immobility time relative to saline-control animals. ACTH animals treated with imipramine, on the other hand, were found to have significantly higher immobility time relative to ACTH-vehicle animals, and saline-imipramine animals. ACTH animals co-administered lithium with imipramine had significantly lower immobility duration relative to saline-vehicle, ACTH-vehicle and ACTH-imipramine groups (see Fig. 3d).

One-way ANOVA also revealed a significant main effect of treatment on *climbing* duration, *F*(4,50) = 17.42, *p* < 0.0001. Shapiro–Wilk normality test was significant for ACTH-imipramine group, *W* = 0.8229, *p* = 0.0188. Tukey’s HSD tests suggest that lithium-treated animals showed significantly longer climbing duration than control-vehicle, ACTH-vehicle, and ACTH-imipramine-treated groups. Saline control animals administered imipramine simlarly showed longer climbing duration than animals in saline-control and ACTH-imipramine groups. ACTH animals administered imipramine alone displayed significantly lower levels of climbing behavior relative to ACTH-control animals (Fig. 3e). No statistically significant main effect of treatment on *swimming* time was observed, one-way ANOVA, *F*(4,50) = 2.221, *p* = 0.0799 (Fig. 3f). As such no post-hoc tests for swimming were conducted. Refer to supplementary tables 1 and 2 for behavioral test descriptives.

### Western blot

The amount of total and phosphorylated Akt, mTOR, and GSK3β in the ILPFC at time of euthanasia (30 min following the FST) was quantified using western blotting, corrected for β-actin, and expressed as a ratio to positive control tissue. Descriptives for Western blot ILPFC proteins are provided in supplementary table 3. The Shapiro–Wilk test was significant for the ACTH group co-administered imipramine and lithium for measures of *p*Akt: *W* = 0.8204, *p* = 0.0471; and *p*GSK3β/GSK3β: *W* = 0.8215, *p* = 0.0358, reflecting minor violations of the assumption of normality.

**Fig. 4:**
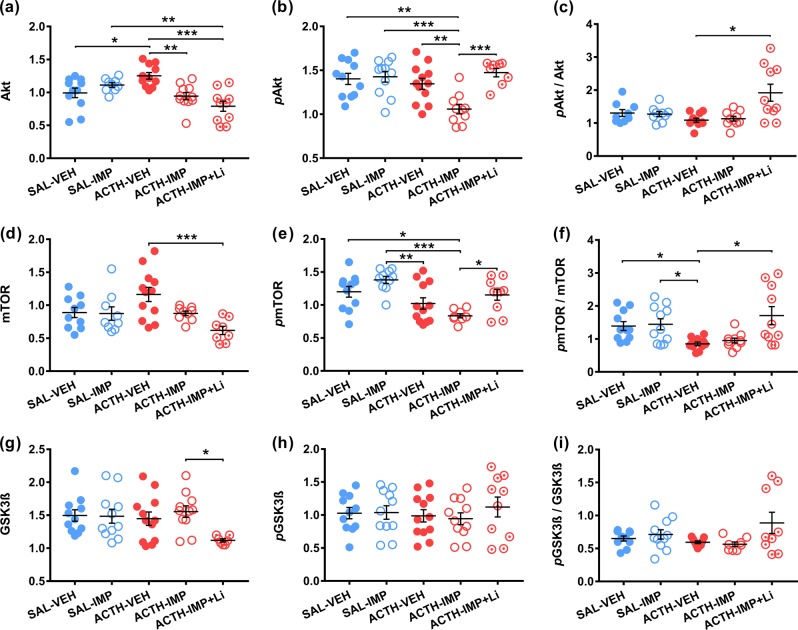
Lithium treatment upregulates insulin signaling in the ILPFC of ACTH animals. Mean levels of total protein, phosphorylated protein, and ratio of phosphorylated:total for Akt (**a**–**c**), mTOR (**d**–**f**), and GSK3β (**g**–**i**) in the ILPFC by treatment group, as measured by Western blot (*n* = 7–12 per group). Data are expressed as mean ± S.E.M; *p < 0.05; **p < 0.01, ***p < 0.001

One-way ANOVA demonstrated a significant main effect of treatment for phosphorylated and total Akt and mTOR (Akt: *F*(4,47) = 8.275, *p* *<* 0.0001; pAkt: *F*(4,47) = 7.105, *p* = 0.0001; mTOR: *F*(4,43) = 5.116, *p* = 0.0018; *p*mTOR: *F*(4,47) = 7.187, *p* = 0.0001). Post-hoc Tukey’s HSD tests multiple comparisons revealed several significant group differences as illustrated in Fig. 4. Some variation in group *n* for protein assays occurred as a result of unviable tissue samples (undetectable protein levels). As a result, these group protein data are depicted as scatter plots.

No significant main effect of treatment was observed for *p*GSK3β: *F*(4,50) = 0.3817, *p* = 0.82; or GSK3β: *F*(4,47) = 2.535, *p* = 0.0524. Closer inspection of GSK3β data however, indicated post-tests were appropriate. Post-tests unveiled a significant reduction in GSK3β for ACTH animals co-administered lithium compared to those that received imipramine alone (*p* = 0.034) (see Fig. 4g).

The Brown–Forsythe test for homogeneity of variance was significant for *p*Akt/Akt: *F*(4,44) = 5.841, *p* < 0.001; *p*mTOR/mTOR, *F*(4,48) = 5.442, *p* = 0.001; and *p*GSK3β/GSK3β: *F*(4,42) = 4.1, *p* = 0.007. Kruskal–Wallis one-way tests indicated significant main effects for pAkt/Akt: *H*(4) = 10.94, *p* = 0.027; *p*mTOR/mTOR: *H*(4) = 16.75, *p* = 0.002; but not *p*GSK3β/GSK3β: *H*(4) = 5.055, *p* = 0.282. Dunn’s post-tests indicated significant group differences for *p*Akt/Akt and *p*mTOR/mTOR, shown in Fig. 4c and f, respectively.

### PBMC insulin challenge

**Fig. 5:**
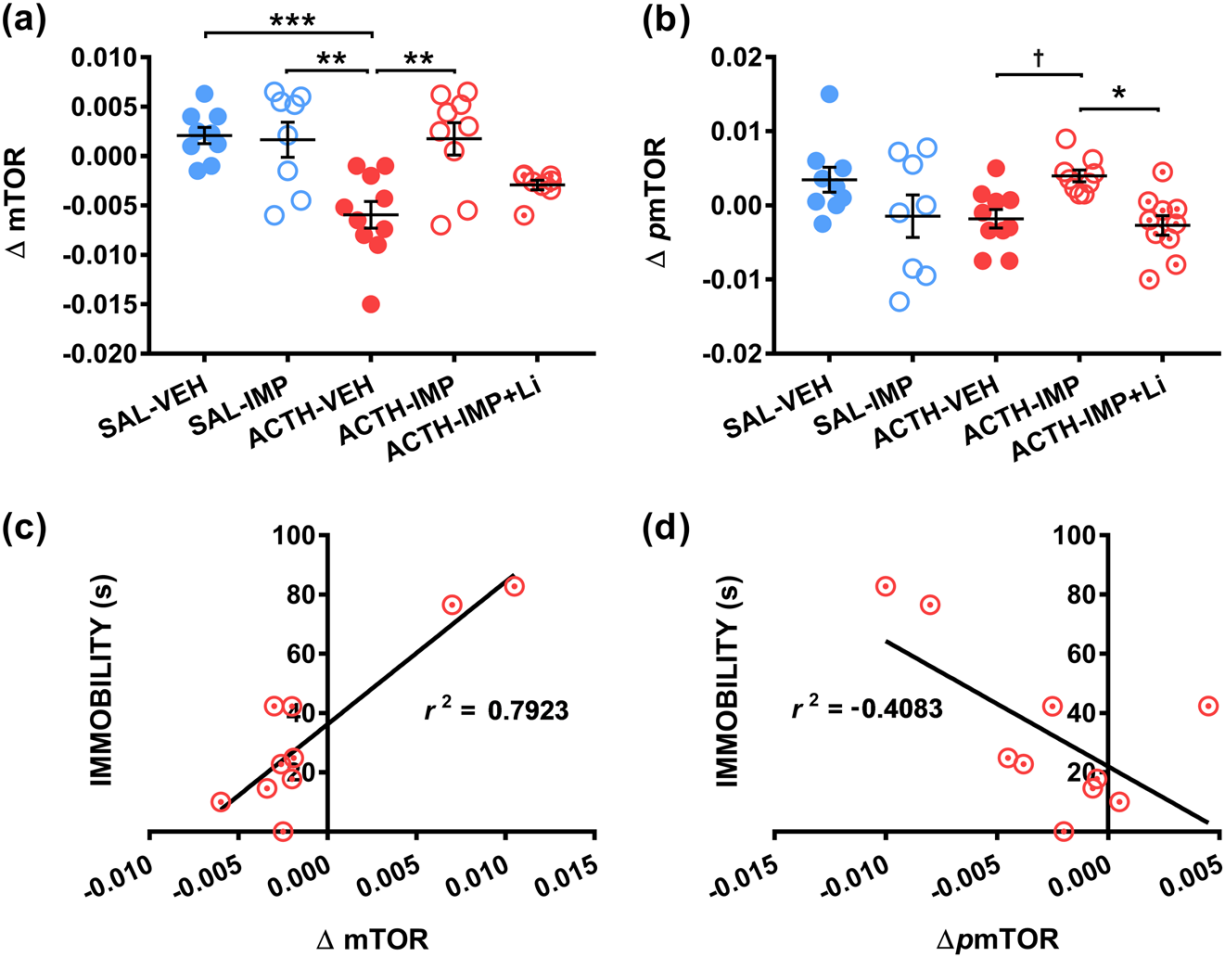
mTOR signaling following insulin challenge differ with treatment, immobility correlated with mTOR activation. The effects of treatment group on the change in (**a**) mTOR and (**b**) *p*mTOR between baseline (*t*_0_) and after 5 min of insulin stimulation (*t*_5_) in PBMCs (*n* = 7–12 per group). Figure also shows the relationship between immobility duration and change in PBMC (**c**) mTOR and (**d**) *p*mTOR following 5 min of insulin stimulation in ACTH pre-treated animals co-administered imipramine (10 mg/kg) and lithium (100 mg/kg), (*n* = 10). Linear regression data are expressed as *r*^2^ values, where significance was assessed via slope regression *F*-tests; ^†^*p* < 0.08; *p < 0.05; **p < 0.01; ***p *<* 0.001

One-way tests were used to evaluate the effects of treatment on change (Δ) in levels of mTOR, and *p*mTOR in PBMCs between baseline (*t*_0_) and after 5 min of insulin stimulation (*t*_5_). One-way ANOVA unveiled a statistically significant main effect of treatment for ΔmTOR, *F*(4,39) = 7.863, *p* < .0001. The Brown–Forsythe test was found to be significant for Δ*p*mTOR, *F*(4, 41) = 3.019, *p* = 0.028. A Kruskal–Wallis one-way test indicated a significant main effect of treatment on Δ*p*mTOR, *H*(4) = 14.46, *p* = 0.006. Subsequent group comparisons utilizing Tukey’s HSD tests and Dunn’s tests unveiled several significant group differences shown in Fig. 5a, b.

Linear regression was subsequently performed on PBMC data to assess the relationship between immobility duration and the reported protein differences and ratios following 5 min of insulin stimulation. For lithium-treated ACTH animals, slope regression tests for both ΔmTOR and Δ*p*mTOR vs. immobility duration were significantly non-zero, *F*(1,8) = 30.52, *p* = 0.0006 and *F*(1,8) = 5.521, *p* = 0.0467, respectively. Immobility duration shared a strong correlation with both ΔmTOR (*r*^2^ = 0.792) and Δ*p*mTOR (*r*^2^ = 0.408). Graphical representation of this relationship is shown in Fig. 5c, d. For mean PBMC protein levels, and slope regression data for all treatment groups, see supplementary tables 4 and 5.

Linear regression was also performed on PBMC data to assess the relationship between protein levels in the ILPFC following exposure to the FST and in PBMCs post-insulin challenge across all animals. Significant positive correlations were observed between *p*mTOR/mTOR levels in the ILPFC and ΔmTOR (*r*^2^ = 0.351) and Δ*p*mTOR (*r*^2^ = −0.314) in PBMCs.

## Discussion

Lithium can improve or augment antidepressant response in some individuals who are otherwise resistant; reportedly reducing recurrence of depressive episodes, lowering suicidality and improving rates of remission^32,33^. Adjunctive lithium treatment is a typical “next-step” after switching to a non-SSRI antidepressant to cater for non-response^4,32^; however, it is only effective for about half of patients with treatment-resistant depression^13,14^. To date, no clear predictive markers for resistance or response to lithium treatment have been uncovered. In this study, we reaffirmed that animals pretreated with ACTH for 14 days are resistant to the acute effects of imipramine (10 mg/kg) in the FST. We also affirmed that acute co-administration of lithium (100 mg/kg) alongside imipramine (10 mg/kg) in these animals rescues the typical antidepressant-like effects of imipramine in the FST. Given the impact of lithium on cellular metabolic processes mediated, in part, by the insulin signaling pathway, we further explored the direct impact of lithium augmentation on insulin signaling in brain and blood tissue.

### Behavioral findings

#### Locomotor effects associated with lithium co-administration

As previously reported, neither ACTH pre-treatment nor imipramine antidepressant administration significantly altered locomotor behavior^6,9^. However, an effect of lithium on locomotor behavior in the OFT was observed. ACTH animals treated with the imipramine–lithium combination showed an overall reduction in distance and mean velocity of travel when compared to both vehicle control-treated saline and ACTH animals. This is less surprising when considering lithium’s anti-manic and mood stabilizing clinical outcomes. Within this context, lithium has been previously described to attenuate exploratory and locomotor-associated behaviors in rodents^34,35^. Notably, previous studies did not directly report on locomotor effects for lithium treatment in their original study undertaken in ACTH pretreated rats^6^.

ACTH animals that received imipramine in conjunction with lithium also exhibited lower time spent in the central region of the OFT, relative to other groups. Typically, low center duration is considered indicative of an anxious phenotype^36^. We propose that this somewhat unexpected anxiety-like effect results instead from the overall impact of lithium on mobility. That is, the reduced time spent in the central region duration may simply reflect a reduction in exploratory behavior corresponding to the aforementioned reduction in locomotor behavior. As well, it is possible that the acute lithium injection may have caused some discomfort through an adverse side-effect, affecting these behaviors (for example, a negative gastrointestinal effect)^37^; however, this is unlikely given the observed increased activity observed in the FST.

#### Lithium restores antidepressant response to imipramine in ACTH pre-treated animals

Using the FST we first sought to affirm that ACTH pre-treatment blocked the antidepressant-like immobility reducing effects of imipramine, as reported previously^5–9^. Imipramine has been observed to reliably reduce immobility in healthy control animals^30^, including those used in our previous research^9^. Consistent with this, we found that administeration of imipramine elicited an antidepressant-like efect in control animals, observed via significantly reduced immobility time in the FST compared to those administered vehicle saline. In contrast, imipramine did not reduce immobility time in ACTH-treated animals. Instead, these animals displayed significantly increased immobility time, coupled with a corresponding decrease in climbing time, compared to ACTH-treated animals administered vehicle saline. As anticipated, when lithium was co-administered with imipramine, a robust antidepressant-like effect was observed in ACTH-treated animals. Specifically, these animals exhibited significantly shorter immobility duration and longer climbing duration than ACTH animals that received imipramine alone, or vehicle saline.

Together, these behavioral data suggest that, consistent with previous findings, ACTH pre-treatment adversely affects the efficacy of imipramine in the FST, promoting antidepressant non-response^9^. Furthermore, these results indicate that lithium co-administration may rescue the typical antidepressant actions of imipramine in ACTH pre-treated animals^6^. Biological correlates for the observed behavioral response may offer some insight into the mechanisms of both ACTH-induced antidepressant treatment resistance, and restoration of therapeutic efficacy via lithium augmentation; this is explored in the next section.

### Insulin signaling upregulated by lithium in ACTH pre-treated rats

#### ILPFC insulin signaling upregulated in ACTH pre-treated rats administered adjunctive lithium

Here we assessed levels of insulin signaling proteins: mTOR, Akt, and GSK3β, as well as *p*Akt, *p*GSK3β, and *p*mTOR in the ILPFC, using Western blot. The ILPFC *p*Akt/Akt ratio was significantly elevated in lithium-treated ACTH animals. ILPFC *p*mTOR/mTOR ratios were significantly lower in ACTH animals administered vehicle saline, and were significantly elevated again in animals co-administered lithium. *p*mTOR and *p*Akt levels were significantly reduced in the ILPFC of ACTH pretreated animals administered imipramine alone; while animals co-administered lithium had similar levels of activation to the control saline groups.

ILPFC GSK3β levels were significantly lower in ACTH animals co-administered lithium and imipramine compared to those that received imipramine alone. While *p*GSK3β levels and pGSK3β/GSK3β ratio were very slightly elevated in lithium-treated animals, this effect was non-significant. As such, the expected pattern of increased phosphorylation (and deactivation) of GSK3β consistent with the purported mechanisms of lithium, was less evident. It is interesting that fewer significant differences or patterns emerged for GSK3β protein levels across treatment conditions, despite previous research establishing that lithium inhibits GSK3β activity, both directly and indirectly^15^. It is important to note that lithium may not be acting exclusively via GSK3β given inhibition of inositol monophosphatase (IMPase) is an additional putative mechanism of lithium action that occurs independent of insulin signaling, yet contributes directly to therapeutic response^38^. Further, other brain regions may show differential insulin signaling responses to lithium with respect to FST outcomes. For example, imipramine co-administered with lithium was previously reported to normalize cell proliferation in the hippocampus of ACTH pretreated animals^39,40^. This effect was similarly associated with FST antidepressant efficacy, yet the effect of lithium on hippocampal GSK3 and/or IMPase function, and its contribution to these effects remains to be determined.

Nevertheless, lithium is known to inhibit GSK3β both directly (via competition with Mg^2+^), and indirectly via Akt^41^, promoting mTOR activation^42^. The observed increased levels of mTOR and Akt phosphorylation in the ILPFC of lithium-treated animals are indeed consistent with insulin signaling pathway activation^16^, and both Akt and mTOR activation have been implicated in treatment response previously^19,25^ making them important candidates for further investigation.

#### Insulin-evoked PBMCs mTOR activation is upregulated by lithium and correlates with antidepressant response in ACTH pre-treated rats

In our final experiment, we isolated PBMCs from each animal to assess if this accessible peripheral tissue could be used as a proxy marker for predicting early treatment response to lithium, with specific respect to lithium’s augmentation of cellular responses to insulin. mTOR activity is affected by various cellular inputs, ranging from whether certain amino acids and growth factors are present, to the energy and nutrient status of the cell^38^. Taking measurements at baseline (*t*_0_) and after 5 min exposure to insulin (*t*_5_), we investigated the effects of treatment condition on levels of mTOR and *p*mTOR in PBMCs.

The observed patterns of protein levels across treatments were somewhat complex. We found significant differences in ΔmTOR in the PBMCs of ACTH-vehicle animals following 5 min of insulin challenge, in contrast to other treatment groups. Change in *p*mTOR levels was significantly lower in the PBMCs of ACTH animals co-treated with imipramine and lithium relative to ACTH animals receiving only imipramine, suggesting possible normalization. Exploring this relationship further, as shown in Fig. 5c, d, linear regression unveiled significant large positive correlations with immobility time and change in mTOR levels. Moderate to large negative correlations were also observed between immobility duration and *p*mTOR. Interestingly, the observed relationship was limited to those animals that received lithium, and not in those that received imipramine alone.

mTOR plays a critical role in integrating intracellular and extracellular signals to regulate cellular metabolism, growth proliferation, and survival^21^, all of which are critical for establishment of effective antidepressant responses and modulated in part by insulin action in the brain. We herein propose that quantificaiton of functional mTOR response to insulin, following acute exposure to lithium treatment, may be one path towards identifying individuals with increased likelihood of achieving a therapeutic response. Given the role that mTOR plays as a cellular sensor responding to energy and stress, modulating synaptogenesis and apoptosis, it is well positioned to serve as an ideal candidate for the evaluation of molecular responses to pharmacotherapies, such as lithium at the cellular level.

### Limitations and concluding remarks

In summary and consistent with previous studies, ACTH pre-treatment was found to block the immobility reducing effects of imipramine (10 mg/kg) in the FST. The effects of imipramine were rescued by the co-administration of lithium (100 mg/kg) in these animals. mTOR and Akt phosphorylation ratios were increased in the ILPFC of lithium-treated animals. Insulin stimulation (10 mg/mL for 5 min) of isolated PBMCs yielded some interesting differences in protein response. ACTH pretreated animals that received imipramine exhibited increased total *p*mTOR activation following insulin challenge. Augmentation with lithium normalized *p*mTOR levels, distinguishing responsive lithium-treated animals from those resistant to imipramine. Moreover, immobility duration was highly correlated with insulin-stimulated mTOR and *p*mTOR levels in lithium-treated animals’ PBMCs. We propose that PBMC insulin challenge may be a useful probe for predicting antidepressant response to lithium, and potentially other therapies.

This study did have some limitations. First, in efforts to minimize animal use, lithium was not examined as a monotherapy, nor was it co-administered in saline-treated animals with imipramine. As such, given that lithium was used as an augmentative treatment in this study, the results presented here should be interpreted with the proviso that imipramine was co-administered with lithium. It should also be noted that acute doses were used, and at the clinical level, both lithium and imipramine typically take several weeks to take effect^43^. It is, however, widely appreciated that the acute administration of a tricyclic antidepressant, such as imipramine reduces the employment of passive coping strategies, such as immobility, and increases active coping behaviors, such as climbing and swimming, in the FST^30^.

As previously discussed, initial research utilizing this model has consistently reported that chronic administration of ACTH renders animals resistant to the therapeutic effects of tricyclic antidepressants (such as imipramine and desipramine) in the FST^5–9^. While some studies have reported ‘depressive-like’ effects of ACTH treatment in this model (e.g.^8^), the majority, this study included, reported no significant increase in immobility duration following ACTH treatment alone^5–7,9^. In this context, the FST should be viewed as a tool for probing antidepressant-like responses, rather than a model of depressive behavior per se. While ACTH model does not exhibit high face validity for depression-like behavior, in contrast to other paradigms, its use of the established predictive validity of the FST makes it useful for assessing antidepressant efficacy. As such, the ACTH model can be utilized to focus specifically on resistance and response to antidepressants.

In this study, we have investigated but a few key proteins in a broad pathway; therefore more in depth investigation of related upstream and downstream targets is warranted for future studies. To our knowledge, this is the first time PBMC insulin-challenge has been used for the purpose of delineating cellular antidepressant response mechanisms, and its utility as a biomarker for brain and behavioral effects that predict treatment response warrants further investigation. First, it would be worthwhile investigating how other brain regions compare in their insulin signaling profile in ACTH animals. For example, the ILPFC and prelimbic PFC are known to have discrete and often contrasting functional roles (e.g.^44^). It would be interesting to investigate whether distinct, or even inverse patterns of insulin signaling emerge across regions dependent on their relative activity during the FST paradigm. Second, it would be most interesting to evaluate whether a similar observation can be made in PBMCs of animals in response to other treatments, especially those dependent on mTOR activation—such as ketamine^19^. We previously reported a divergent response to ketamine (10 mg/kg) in FST for ACTH animals, deemed responders/non-responders^45^; in a differentiable cohort such as this we could appropriately assess whether PBMC mTOR challenge might predict FST behaviors. Finally, it is worth mentioning the significant clinical potential of this assay. In future, clinical researchers might assess whether findings such as these translate to patients receiving lithium augmentation therapy.

## Supplementary information

Supplementary Tables
