# Supplementary material for: Insulin-stimulated mTOR activation in peripheral blood mononuclear cells associated with early treatment response to lithium augmentation in rodent model of antidepressant-resistance
Source: Transl Psychiatry. 2019 Mar 15;9:113. doi: 10.1038/s41398-019-0434-5 (PMC6420640; doi:10.1038/s41398-019-0434-5)
Supplement: Supplementary file 1 — Supplementary Tables [file 41398_2019_434_MOESM1_ESM.docx]

| Supplementary table 1. | | | | | | | | | | | | | | | | | | | | |
| --- | --- | --- | --- | --- | --- | --- | --- | --- | --- | --- | --- | --- | --- | --- | --- | --- | --- | --- | --- | --- |
| *Descriptive statistics for OFT behaviors* | | | | | | | | | | | | | | | | | | | | |
|  |  | Treatment | | | | | | | | | | | | | | | | | | |
|  |  | Saline | | | | | | |  | ACTH | | | | | | | | | | |
|  |  | Saline | | |  | Imipramine | | |  | Saline | | |  | Imipramine | | |  | Imipramine+Lithium | | |
| Behavior |  | *mean* | *S.E.M* | *n* |  | *mean* | *S.E.M* | *n* |  | *mean* | *S.E.M* | *n* |  | *mean* | *S.E.M* | *n* |  | *mean* | *S.E.M* | *n* |
| *Distance* |  | 27.31 | 0.34 | 11 |  | 24.96 | 1.56 | 11 |  | 28.16 | 0.92 | 12 |  | 24.66 | 0.73 | 11 |  | 19.36 | 1.96 | 10 |
| *Velocity* |  | 75.92 | 0.92 | 11 |  | 69.48 | 4.36 | 11 |  | 78.28 | 2.56 | 12 |  | 68.66 | 2.05 | 11 |  | 53.79 | 5.45 | 10 |
| *Center duration* |  | 40.59 | 2.09 | 11 |  | 44.30 | 5.17 | 11 |  | 39.48 | 3.82 | 12 |  | 43.58 | 2.18 | 11 |  | 24.35 | 2.41 | 10 |
|  | | | | | | | | | | | | | | | | | | | | |

| Supplementary table 2. | | | | | | | | | | | | | | | | | | | | |
| --- | --- | --- | --- | --- | --- | --- | --- | --- | --- | --- | --- | --- | --- | --- | --- | --- | --- | --- | --- | --- |
| *Descriptive statistics for FST behaviors* | | | | | | | | | | | | | | | | | | | | |
|  |  | Treatment | | | | | | | | | | | | | | | | | | |
|  |  | Saline | | | | | | |  | ACTH | | | | | | | | | | |
|  |  | Saline | | |  | Imipramine | | |  | Saline | | |  | Imipramine | | |  | Imipramine+Lithium | | |
| Behavior |  | *mean* | *S.E.M* | *n* |  | *mean* | *S.E.M* | *n* |  | *mean* | *S.E.M* | *n* |  | *mean* | *S.E.M* | *n* |  | *mean* | *S.E.M* | *n* |
| *Immobility* |  | 74.97 | 7.37 | 10 |  | 40.86 | 4.58 | 11 |  | 66.69 | 7.37 | 12 |  | 95.39 | 8.16 | 11 |  | 26.15 | 7.05 | 10 |
| *Climbing* |  | 116.50 | 7.02 | 11 |  | 152.90 | 6.96 | 11 |  | 135.80 | 7.84 | 12 |  | 103.50 | 5.18 | 11 |  | 183.70 | 9.46 | 10 |
| *Swimming* |  | 53.15 | 5.46 | 11 |  | 46.20 | 6.31 | 11 |  | 37.47 | 6.38 | 12 |  | 41.13 | 5.08 | 11 |  | 30.11 | 5.04 | 10 |
|  |  |  |  |  |  |  |  |  |  |  |  |  |  |  |  |  |  |  |  |  |
|  |  |  |  |  |  |  |  |  |  |  |  |  |  |  |  |  |  |  |  |  |

| Supplementary table 3. | | | | | | | | | | | | | | | | | | | | |
| --- | --- | --- | --- | --- | --- | --- | --- | --- | --- | --- | --- | --- | --- | --- | --- | --- | --- | --- | --- | --- |
| *Mean levels of insulin signaling pathway proteins in ILPFC* | | | | | | | | | | | | | | | | | | | | |
|  |  | Treatment | | | | | | | | | | | | | | | | | | |
|  |  | Saline | | | | | | |  | ACTH | | | | | | | | | | |
|  |  | Saline | | |  | Imipramine | | |  | Saline | | |  | Imipramine | | |  | Imipramine+Lithium | | |
| Protein |  | *mean* | *S.E.M* | *n* |  | *mean* | *S.E.M* | *n* |  | *mean* | *S.E.M* | *n* |  | *mean* | *S.E.M* | *n* |  | *mean* | *S.E.M* | *n* |
| Akt |  | 0.99 | 0.07 | 11 |  | 1.11 | 0.03 | 9 |  | 1.25 | 0.05 | 11 |  | 0.94 | 0.05 | 11 |  | 0.79 | 0.08 | 10 |
| *p*Akt |  | 1.40 | 0.06 | 11 |  | 1.43 | 0.06 | 11 |  | 1.35 | 0.06 | 12 |  | 1.06 | 0.05 | 10 |  | 1.47 | 0.05 | 8 |
| *p*Akt/Akt |  | 1.31 | 0.10 | 9 |  | 1.27 | 0.08 | 9 |  | 1.09 | 0.06 | 11 |  | 1.13 | 0.07 | 10 |  | 1.92 | 0.26 | 10 |
|  |  |  |  |  |  |  |  |  |  |  |  |  |  |  |  |  |  |  |  |  |
| mTOR |  | 0.89 | 0.08 | 10 |  | 0.87 | 0.10 | 9 |  | 1.16 | 0.11 | 12 |  | 0.88 | 0.03 | 9 |  | 0.62 | 0.06 | 8 |
| *p*mTOR |  | 1.20 | 0.08 | 11 |  | 1.38 | 0.05 | 10 |  | 1.02 | 0.09 | 12 |  | 0.84 | 0.03 | 9 |  | 1.15 | 0.08 | 10 |
| *p*mTOR/mTOR |  | 1.39 | 0.13 | 11 |  | 1.45 | 0.17 | 11 |  | 0.85 | 0.05 | 11 |  | 0.95 | 0.07 | 10 |  | 1.71 | 0.27 | 10 |
|  |  |  |  |  |  |  |  |  |  |  |  |  |  |  |  |  |  |  |  |  |
| GSK3β |  | 1.50 | 0.09 | 11 |  | 1.49 | 0.11 | 11 |  | 1.45 | 0.10 | 12 |  | 1.56 | 0.09 | 11 |  | 1.12 | 0.02 | 7 |
| *p*GSK3β |  | 1.03 | 0.09 | 11 |  | 1.04 | 0.10 | 11 |  | 0.99 | 0.09 | 12 |  | 0.94 | 0.09 | 11 |  | 1.12 | 0.15 | 10 |
| *p*GSK3β/GSK3β |  | 0.65 | 0.04 | 10 |  | 0.71 | 0.07 | 11 |  | 0.59 | 0.02 | 9 |  | 0.57 | 0.03 | 8 |  | 0.89 | 0.16 | 9 |
|  |  |  |  |  |  |  |  |  |  |  |  |  |  |  |  |  |  |  |  |  |
|  | | | | | | | | | | | | | | | | | | | | |

| Supplementary table 4. | | | | | | | | | | | | | | | | | | | | |
| --- | --- | --- | --- | --- | --- | --- | --- | --- | --- | --- | --- | --- | --- | --- | --- | --- | --- | --- | --- | --- |
| *Mean level of mTOR signaling proteins following PBMC insulin challenge.* | | | | | | | | | | | | | | | | | | | | |
|  |  | Treatment | | | | | | | | | | | | | | | | | | |
|  |  | Saline | | | | | | |  | ACTH | | | | | | | | | | |
|  |  | Saline | | |  | Imipramine | | |  | Saline | | |  | Imipramine | | |  | Imipramine+Lithium | | |
| Protein |  | *mean* | *S.E.M* | *n* |  | *mean* | *S.E.M* | *n* |  | *mean* | *S.E.M* | *n* |  | *mean* | *S.E.M* | *n* |  | *mean* | *S.E.M* | *n* |
| mTOR |  | 0.0021 | 0.0008 | 9 |  | 0.0017 | 0.0018 | 8 |  | -0.0059 | 0.0014 | 10 |  | 0.0018 | 0.0016 | 9 |  | -0.0029 | 0.0005 | 8 |
| *pmTOR* |  | 0.0035 | 0.0017 | 9 |  | -0.0014 | 0.0029 | 8 |  | -0.0018 | 0.0013 | 10 |  | 0.0040 | 0.0008 | 9 |  | -0.0027 | 0.0013 | 10 |
|  | | | | | | | | | | | | | | | | | | | | |

| Supplementary table 5. | | | | | | | | | | |
| --- | --- | --- | --- | --- | --- | --- | --- | --- | --- | --- |
| *Linear regression for immobility duration versus mTOR signaling proteins following PBMC insulin challenge.* | | | | | | | | | | |
|  |  |  | mTOR | | |  | *p*mTOR | | |  |
| Treatment | |  | *r^2^* | *F* | *p* |  | *r^2^* | *F* | *p* |  |
| Saline |  |  |  |  |  |  |  |  |  |  |
|  | Saline |  | 0.0068 | F_(1,7)=_0.0500 | 0.8329 |  | 0.0368 | F_(1,7)=_0.2673 | 0.6211 |  |
|  | Imipramine |  | 0.0432 | F_(1,6)=_0.2707 | 0.6215 |  | 0.0597 | F_(1,6)=_0.3812 | 0.5596 |  |
| ACTH |  |  |  |  |  |  |  |  |  |  |
|  | Saline |  | 0.0178 | F_(1,8)=_0.1415 | 0.7132 |  | 0.0945 | F_(1,8)=_0.8351 | 0.3875 |  |
|  | Imipramine |  | 0.0712 | F_(1,7)=_0.5366 | 0.4877 |  | 0.0756 | F_(1,7)=_0.5726 | 0.4739 |  |
|  | Imipramine+Lithium |  | 0.7923 | F_(1,8)=_30.52 | 0.0006 | *** | 0.4083 | F_(1,8)=_5.521 | 0.0467 | * |
|  | | | | | | | | | | |
|  | | | | | | | | | | |
